# Supplementary material for: Decreased community-acquired pneumonia coincided with rising awareness of precautions before governmental containment policy in Japan
Source: PNAS Nexus. 2023 May 9;2(5):pgad153. doi: 10.1093/pnasnexus/pgad153 (PMC10208112; doi:10.1093/pnasnexus/pgad153)
Supplement: pgad153_Supplementary_Data [file pgad153_supplementary_data.pdf]

Decreased hospitalizations and deaths from community-acquired pneumonia coincided with rising public awareness of personal precautions before the governmental containment and closure policy: A nationwide observational study in Japan

Masato Tashiro[1,2]*, Shuntaro Sato[3]*, Akira Endo[4,5]*, Ryosuke Hamashima[1,6], Yuya Ito[7], Nobuyuki Ashizawa[2,7], Kazuaki Takeda[7], Naoki Iwanaga[7], Shotaro Ide[8], Ayumi Fujita[2], Takahiro Takazono[1,7], Kazuko Yamamoto[7], Takeshi Tanaka[2], Akitsugu Furumoto[8], Katsunori Yanagihara[9], Hiroshi Mukae[7], Kiyohide Fushimi[10], Koichi Izumikawa[1,2]

*Joint first authors

[1]Department of Infectious Diseases, Nagasaki University Graduate School of Biomedical Sciences, Nagasaki, Japan; [2]Infection Control and Education Center, Nagasaki University Hospital, Nagasaki, Japan; [3]Clinical Research Center, Nagasaki University Hospital, Nagasaki, Japan; [4]Department of Infectious Disease Epidemiology, London School of Hygiene & Tropical Medicine, London, United Kingdom; [5]School of Tropical Medicine and Global Health, Nagasaki University, Nagasaki, Japan; [6]Department of Pulmonary Medicine, Graduate School of Medical Science, Kyoto Prefectural University of Medicine, Kyoto, Japan; [7]Department of Respiratory Medicine, Nagasaki University Hospital, Nagasaki, Japan; [8]Infectious Diseases Experts Training Center, Nagasaki University Hospital, Nagasaki, Japan; [9]Department of Laboratory Medicine, Nagasaki University Hospital, Nagasaki, Japan; [10]Department of Health Policy and Informatics, Graduate School of Medical and Dental Sciences, Tokyo Medical and Dental University, Tokyo, Japan.

Corresponding author:

Masato Tashiro, M.D., Ph.D., Department of Infectious Diseases, Nagasaki University Graduate School of Biomedical Sciences, 1-7-1 Sakamoto, Nagasaki 852-8501, Japan

Phone: +81-95-819-7731; Fax: +81-95-849-7766; E-mail: mtashiro@nagasaki-u.ac.jp

SUPPLEMENTAL MATERIAL

SUPPLEMENTAL METHODS

Time trends in indicators of personal precautions and physical contacts

We collected newspaper article counts, mask and hand sanitizer sales data, and Google community mobility data to assess changes in public awareness and behaviors in the population in early 2020. As a measure of public awareness, we obtained the number of articles in two Japanese national newspapers containing specific keywords from January 2018 to August 2020 from their article databases.[1,2] Search keywords related to personal precautions included "masks" and "hand sanitizer" and those related to physical contact included "social distance", "3Cs", and "remote work".[3] All Japanese keywords used in the search and their English translations can be found in the below table. Using the average monthly number of articles between January 2018 and December 2019 as a baseline, we plotted the relative change in the number of articles in Figure 2 in the main text.

	Original search formula in Japanese	English translations*
Personal precautions	"マスク" OR "咳エチケット" OR "手洗い" OR "石鹸" OR "石けん" OR "手指消毒" OR "手指衛生"	“masks” OR “cough etiquette” OR “hand washing” OR “soap” OR “hand sanitizer” OR “hand hygiene”
Physical contacts	"ソーシャルディスタンス" OR "社会的距離" OR "身体的距離" OR "3 密" OR "三密" OR "自粛" OR "自宅" OR "リモートワーク" OR "テレワーク" OR "オンライン会議" OR "時差通勤" OR "自宅勤務" OR "在宅勤務" OR "遠隔勤務" OR "休校" OR "学校休業"	“social distance” OR “physical distance” OR “3Cs” OR “voluntary restraint” OR “home” OR “remote work” OR “telework” OR “online meeting” OR “staggered commuting” OR “work at home” OR “school closure”

* Duplicates are removed where multiple Japanese search terms translate into the same English.

As measures of observed behaviors, we also collected mask and hand sanitizer sales data (personal precautions) and Google community mobility data (physical contacts). Sales data was retrieved from a database provided by INTAGE Inc (Tokyo, Japan) from January 2018 through August 2020. Google community mobility data provides regional-level movement trends by location type (retail and recreation, grocery and pharmacy, parks, transit stations, workplaces, and residential) as a relative change from the day-of-the-week-wise average from January 3 through February 6, 2020. We obtained the daily national average of Google mobility in Japan in all location types but residential to measure the contact behavior of individuals outside the home.

TABLE S1. A detailed summary of Charlson comorbidity index categories

Charlson comorbidity Index	Pneumonia N = 415,712	Pyelonephritis N = 77,924	Biliary tract infections N = 150,528
Myocardial Infarction	7,600 (1.8%)	1,079 (1.4%)	2,817 (1.9%)
Congestive Heart Failure	75,161 (18%)	5,821 (7.5%)	8,846 (5.9%)
Peripheral Vascular Disease	7,645 (1.8%)	1,112 (1.4%)	2,141 (1.4%)
Cerebrovascular Disease	65,710 (16%)	9,104 (12%)	12,926 (8.6%)
Dementia	74,991 (18%)	10,012 (13%)	11,779 (7.8%)
Chronic Pulmonary Disease	74,804 (18%)	3,164 (4.1%)	5,699 (3.8%)
Connective Tissue Disease-Rheumatic Disease	12,791 (3.1%)	2,042 (2.6%)	1,710 (1.1%)
Peptic Ulcer Disease	13,900 (3.3%)	2,361 (3.0%)	9,247 (6.1%)
Mild Liver Disease	11,746 (2.8%)	2,498 (3.2%)	10,159 (6.7%)
Diabetes without complications	60,814 (15%)	12,936 (17%)	26,381 (18%)
Diabetes with complications	14,374 (3.5%)	3,697 (4.7%)	4,114 (2.7%)
Paraplegia and Hemiplegia	1,724 (0.4%)	350 (0.4%)	308 (0.2%)
Renal Disease	24,401 (5.9%)	4,709 (6.0%)	5,193 (3.4%)
Cancer	47,295 (11%)	9,467 (12%)	28,344 (19%)
Moderate or Severe Liver Disease	535 (0.1%)	114 (0.1%)	851 (0.6%)
Metastatic Carcinoma	8,286 (2.0%)	1,486 (1.9%)	4,503 (3.0%)
AIDS/HIV	139 (< 0.1%)	17 (< 0.1%)	15 (< 0.1%)

FIGURE S1. Interrupted time series subanalyses of the number of monthly hospitalizations and 30-day deaths in Japan between April 2015 and August 2020.

Blue lines, red lines, and dots indicate the modeled curve fitted to the observed data, the counterfactual curve assuming no change point in February 2020, and the observed data, respectively. Ribbons indicate 95% confidence intervals. Gray-shaded areas indicate months at the change point (February 2020) and onward. The figures show data from January 2018 but estimates are based on data from April 2015. The mapping between figures and details of the subanalyses is shown in the table below.

Figure label	Outcome	Disease	Age, years	Pneumonia type
A	The number of hospitalizations	Pneumonia	≧ 18	
B		Pyelonephritis	≧ 18	
C		Biliary tract infections	≧ 18	
D		Pneumonia	18-64	
E			≧ 65	
F		Pneumonia	18-64	Pneumococcal pneumonia
G				Aspiration pneumonia
H				Influenza pneumonia
I		Pneumonia	≧ 65	Pneumococcal pneumonia
J				Aspiration pneumonia
K				Influenza pneumonia
L	The number of 30-day deaths	Pneumonia	≧ 18	
M		Pyelonephritis	≧ 18	
N		Biliary tract infections	≧ 18	
O		Pneumonia	18-64	
P			≧ 65	
Q		Pneumonia	18-64	Pneumococcal pneumonia
R				Aspiration pneumonia
S		Pneumonia	≧ 65	Pneumococcal pneumonia
T				Aspiration pneumonia

Outcome: The number of hospitalizations

A

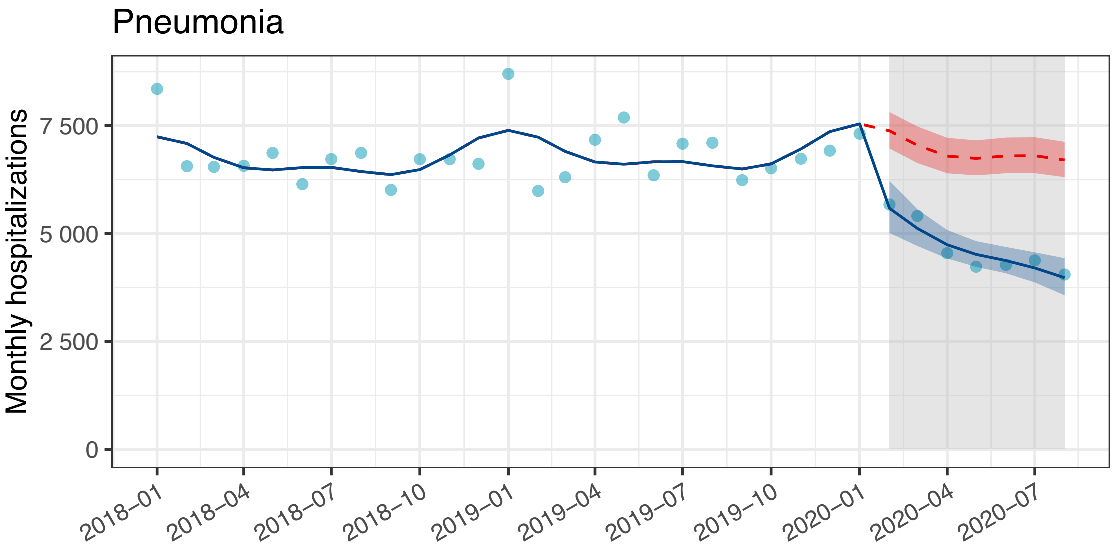

B

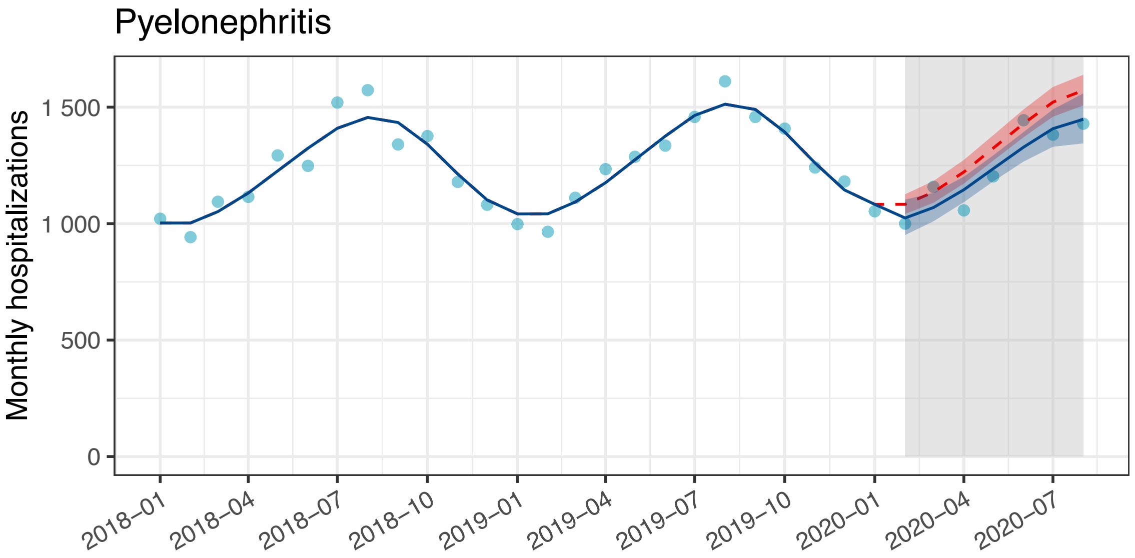

C

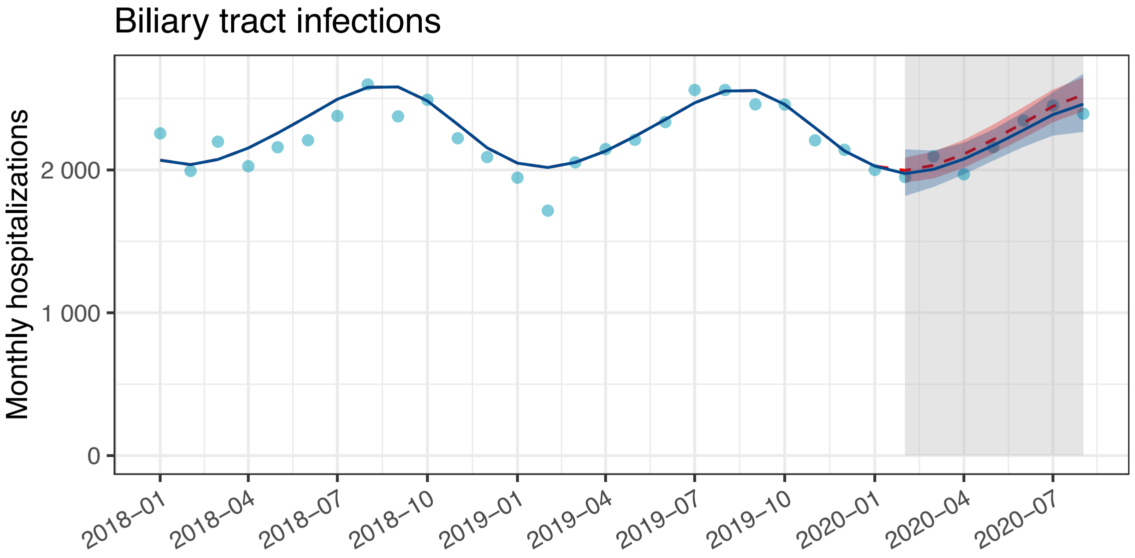

D

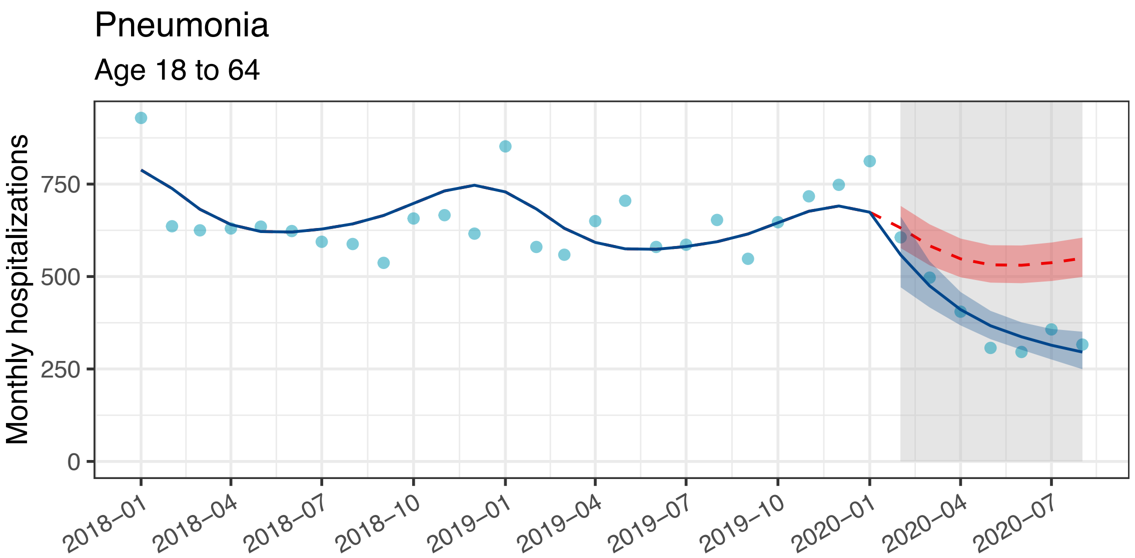
Pneumonia
Age 18 to 64
Monthly hospitalizations
0
250
500
750
2018–01
2018–04
2018–07
2018–10
2019–01
2019–04
2019–07
2019–10
2020–01
2020–04
2020–07

E

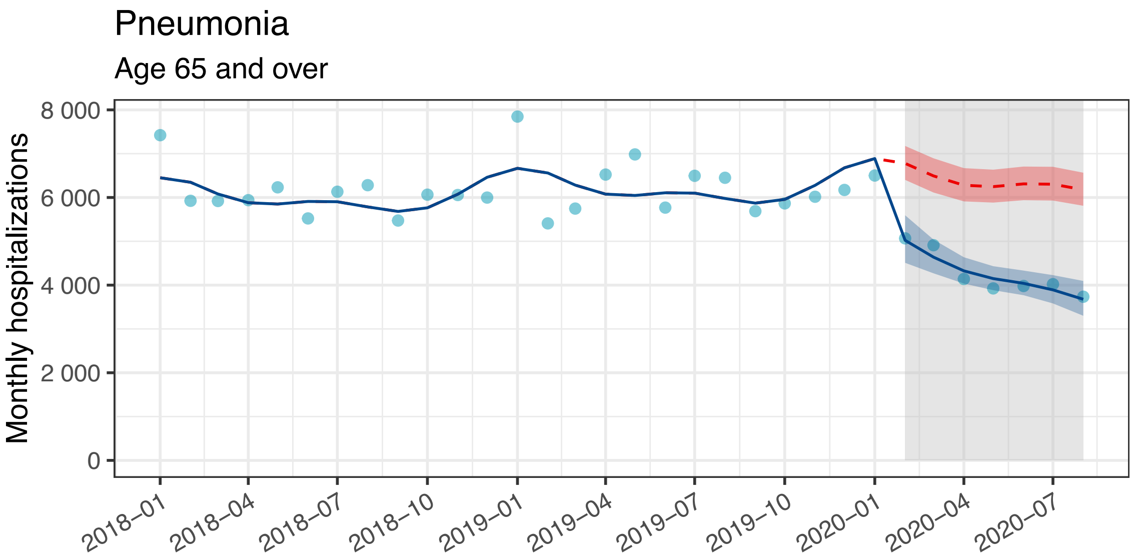
Pneumonia
Age 65 and over
Monthly hospitalizations
0
2 000
4 000
6 000
8 000
2018–01
2018–04
2018–07
2018–10
2019–01
2019–04
2019–07
2019–10
2020–01
2020–04
2020–07

F

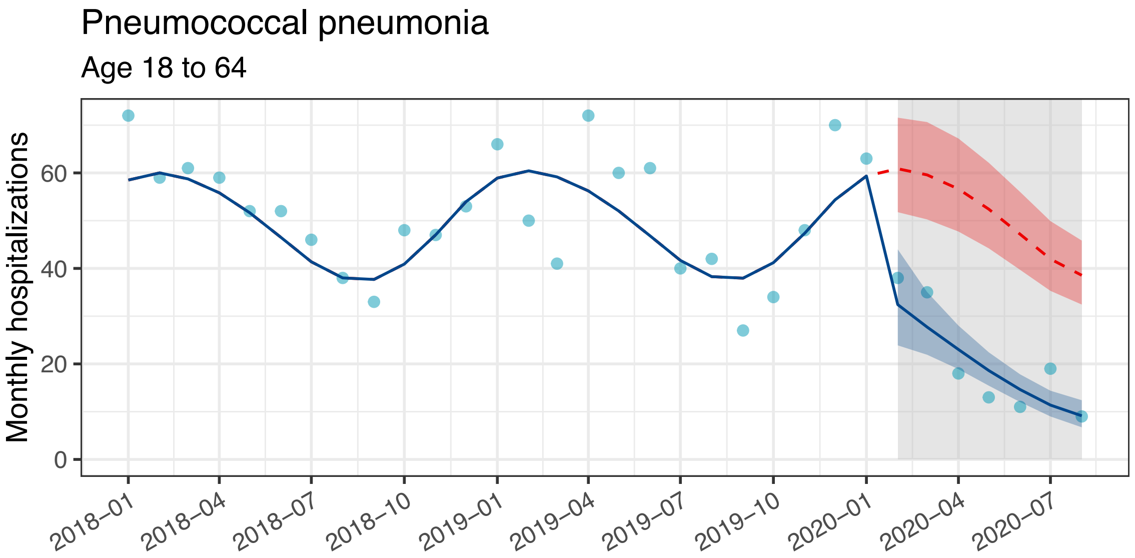
Pneumococcal pneumonia
Age 18 to 64
Monthly hospitalizations
0
20
40
60
2018–01
2018–04
2018–07
2018–10
2019–01
2019–04
2019–07
2019–10
2020–01
2020–04
2020–07

G

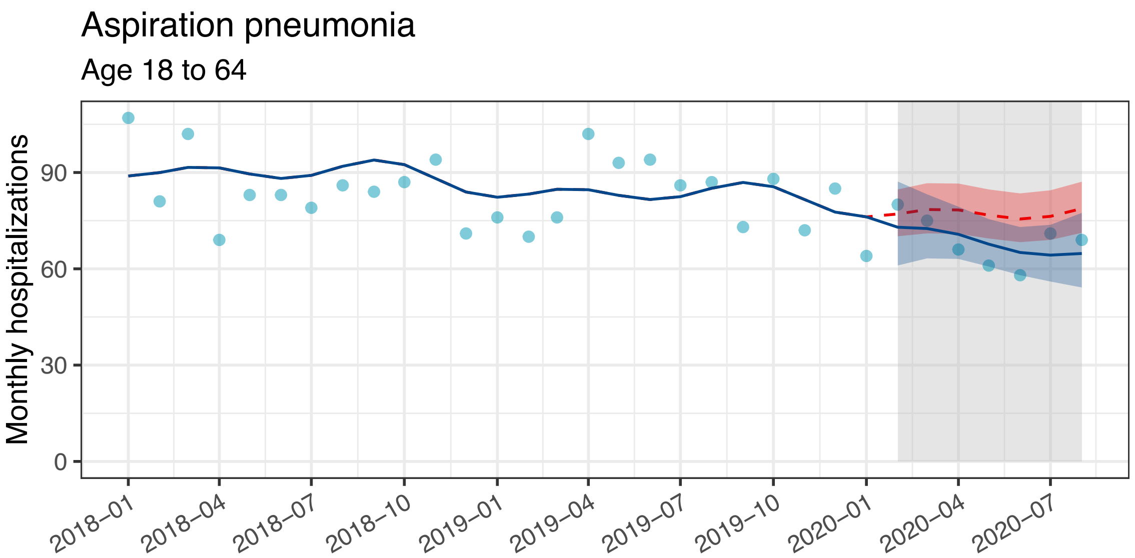
Aspiration pneumonia
Age 18 to 64
Monthly hospitalizations
90
60
30
0
2018–01
2018–04
2018–07
2018–10
2019–01
2019–04
2019–07
2019–10
2020–01
2020–04
2020–07

H

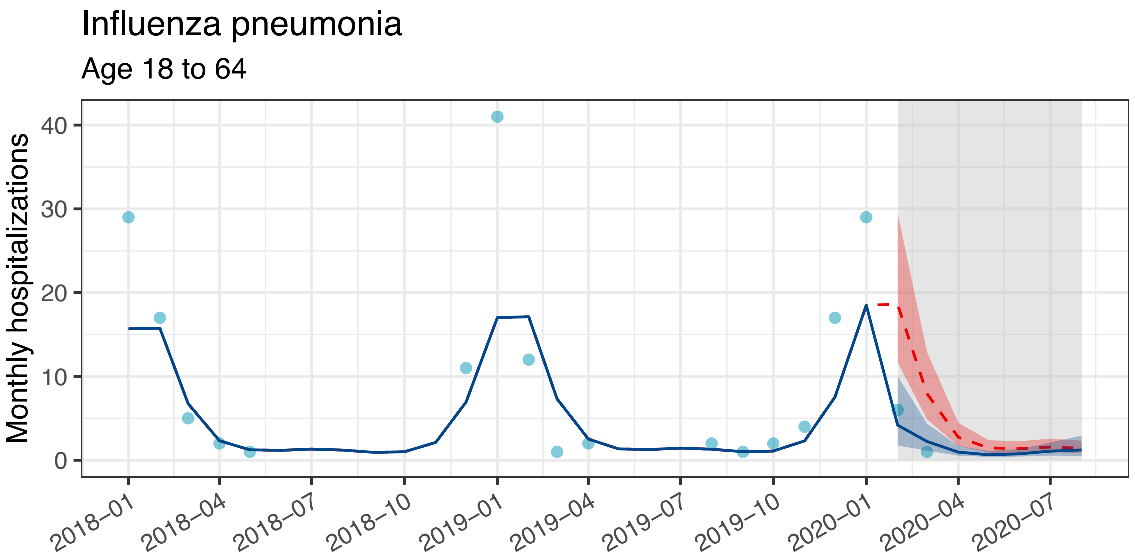
Influenza pneumonia
Age 18 to 64
Monthly hospitalizations
40
30
20
10
0
2018–01
2018–04
2018–07
2018–10
2019–01
2019–04
2019–07
2019–10
2020–01
2020–04
2020–07

I

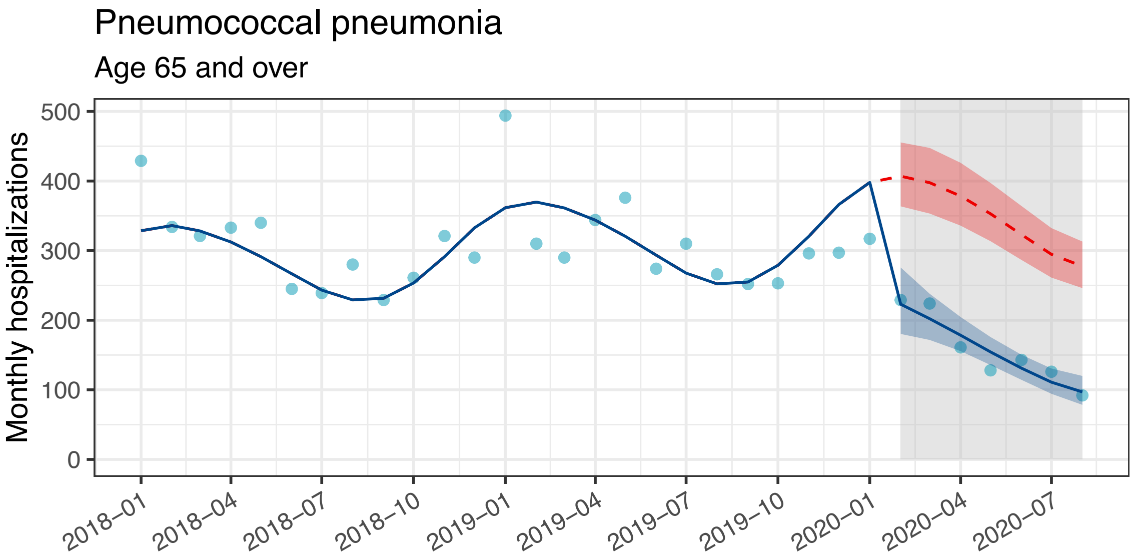
Pneumococcal pneumonia
Age 65 and over
Monthly hospitalizations
500
400
300
200
100
0
2018–01
2018–04
2018–07
2018–10
2019–01
2019–04
2019–07
2019–10
2020–01
2020–04
2020–07

J

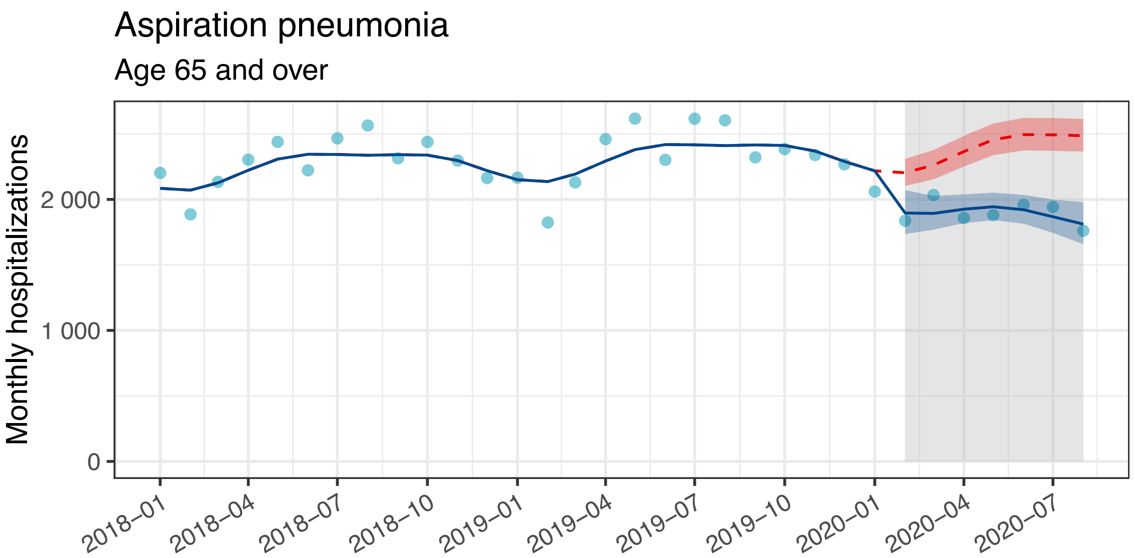
Aspiration pneumonia
Age 65 and over
Monthly hospitalizations
2 000
1 000
0
2018–01
2018–04
2018–07
2018–10
2019–01
2019–04
2019–07
2019–10
2020–01
2020–04
2020–07

K

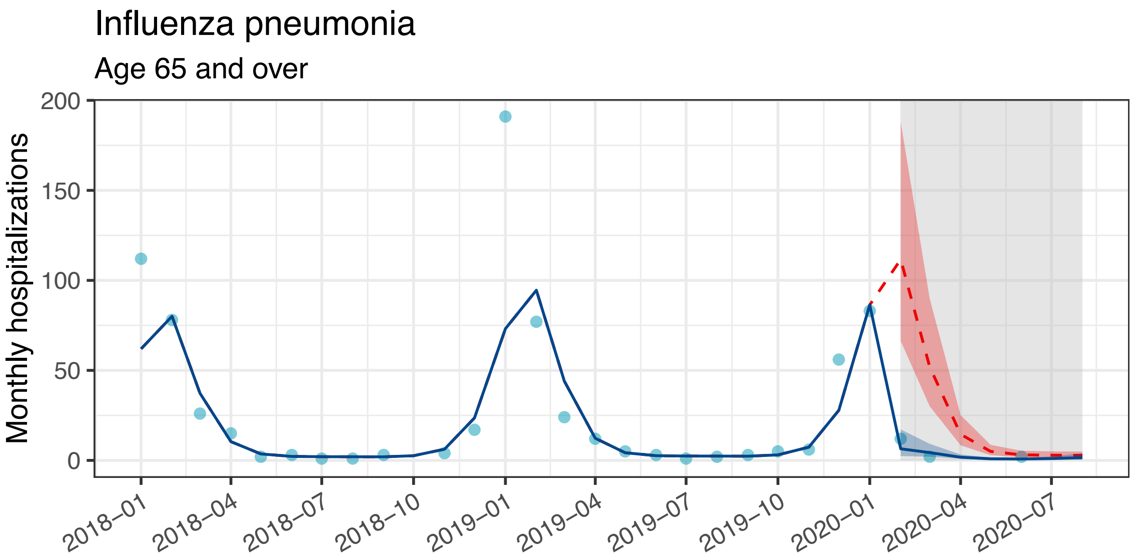
Influenza pneumonia
Age 65 and over
Monthly hospitalizations
200
150
100
50
0
2018–01
2018–04
2018–07
2018–10
2019–01
2019–04
2019–07
2019–10
2020–01
2020–04
2020–07

Outcome: The number of 30-day deaths

L

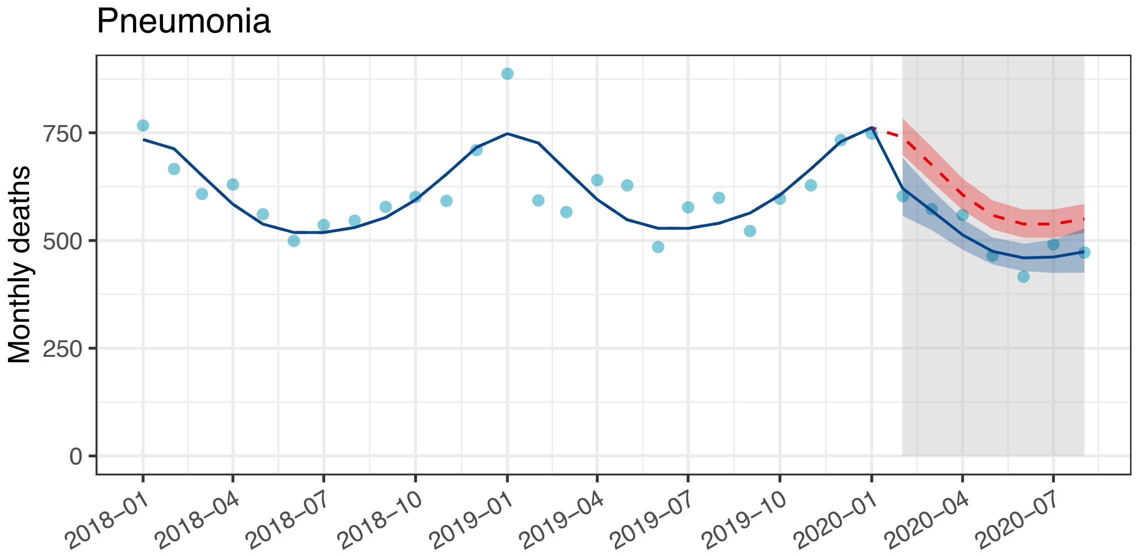

M

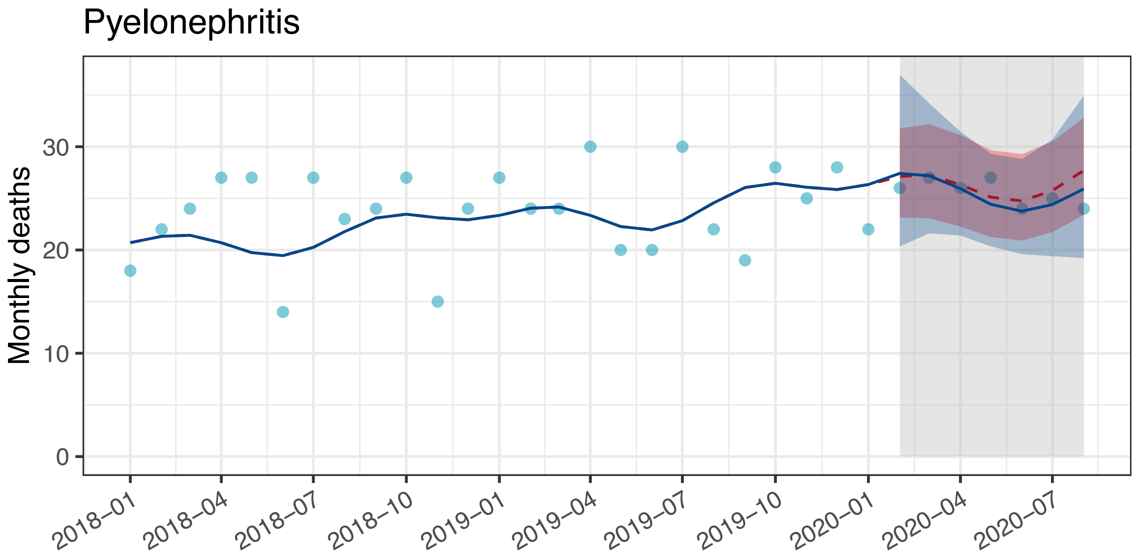

N

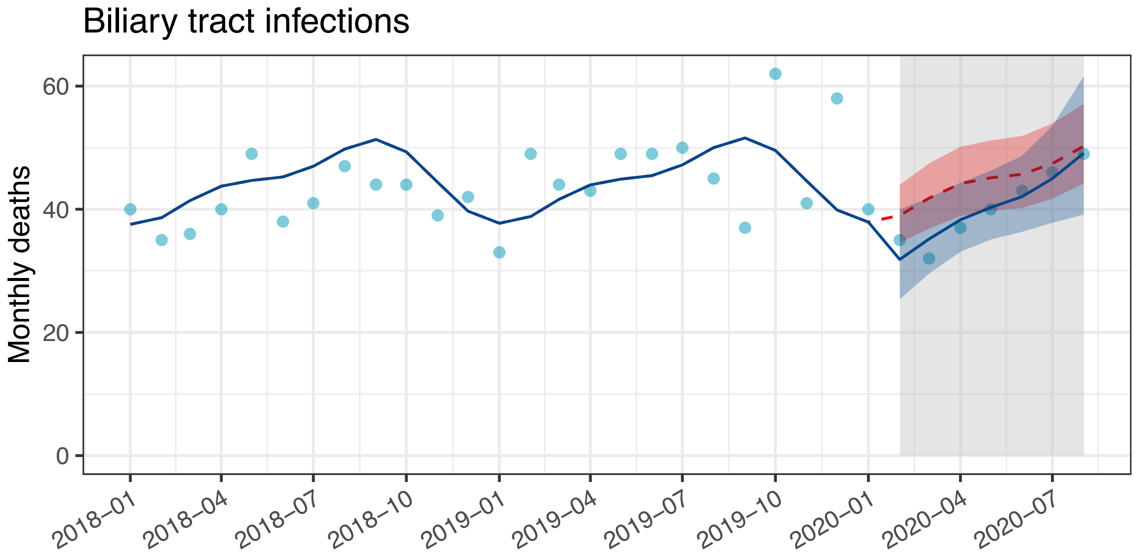

O

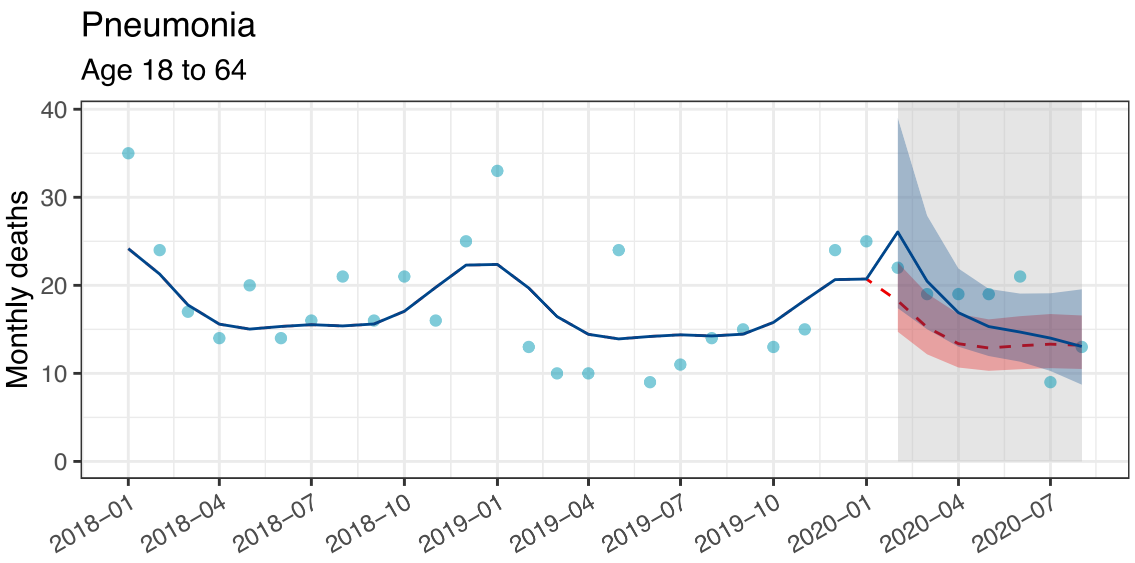
Pneumonia
Age 18 to 64
Monthly deaths
0
10
20
30
40
2018–01
2018–04
2018–07
2018–10
2019–01
2019–04
2019–07
2019–10
2020–01
2020–04
2020–07

P

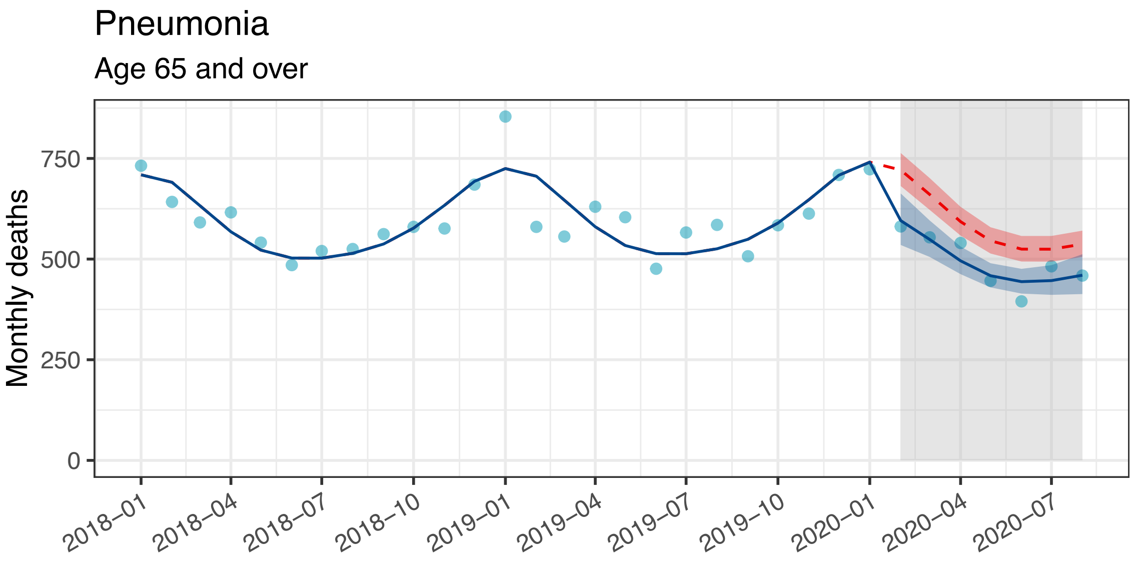
Pneumonia
Age 65 and over
Monthly deaths
0
250
500
750
2018–01
2018–04
2018–07
2018–10
2019–01
2019–04
2019–07
2019–10
2020–01
2020–04
2020–07

Q

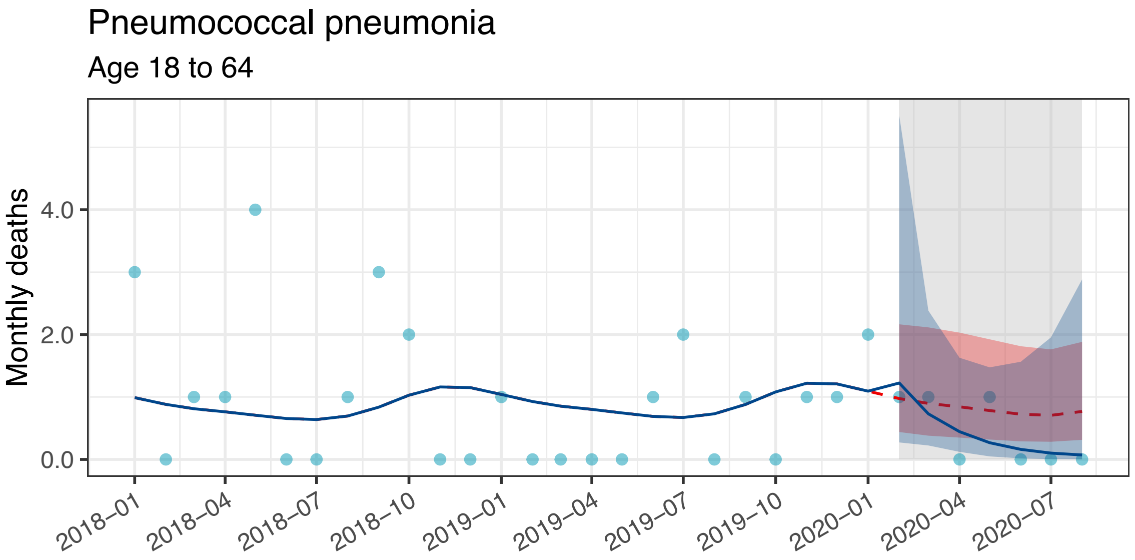
Pneumococcal pneumonia
Age 18 to 64
Monthly deaths
0.0
2.0
4.0
2018–01
2018–04
2018–07
2018–10
2019–01
2019–04
2019–07
2019–10
2020–01
2020–04
2020–07

R

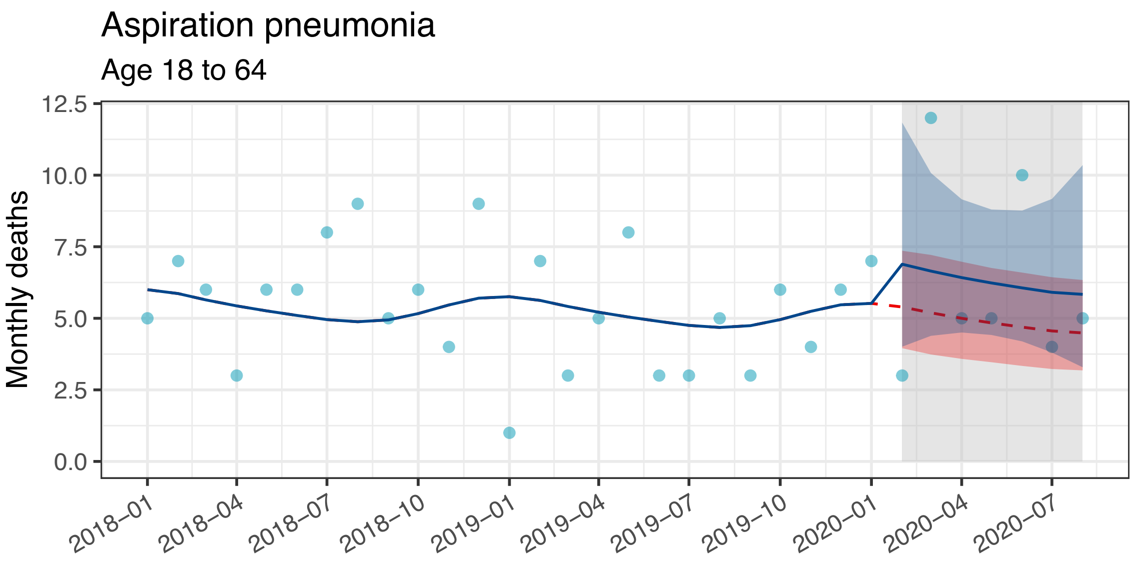

Aspiration pneumonia
Age 18 to 64
Monthly deaths
12.5
10.0
7.5
5.0
2.5
0.0
2018–01
2018–04
2018–07
2018–10
2019–01
2019–04
2019–07
2019–10
2020–01
2020–04
2020–07

S

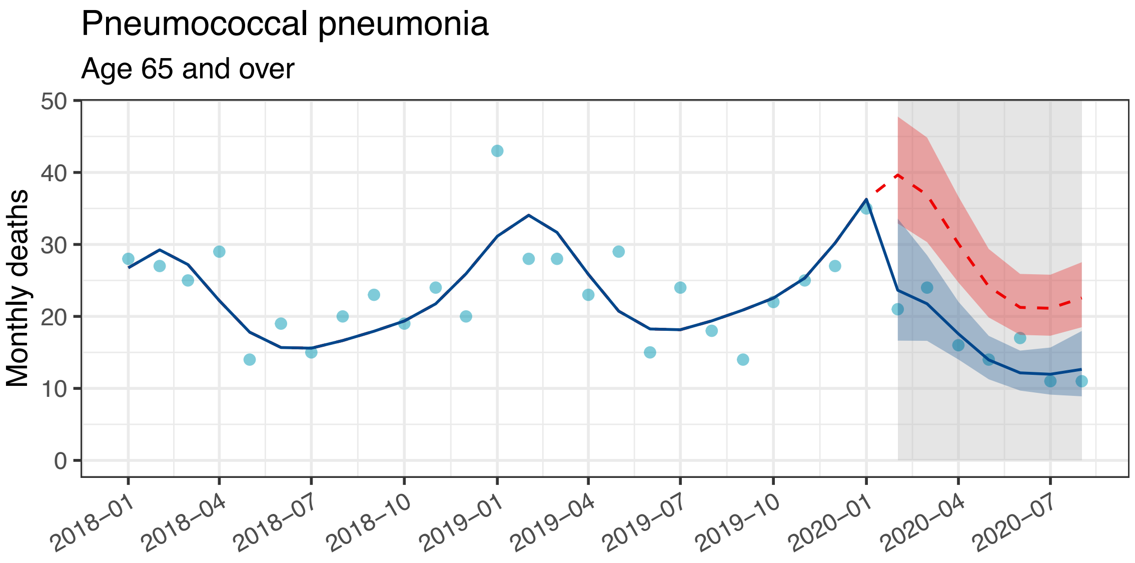

Pneumococcal pneumonia
Age 65 and over
Monthly deaths
50
40
30
20
10
0
2018–01
2018–04
2018–07
2018–10
2019–01
2019–04
2019–07
2019–10
2020–01
2020–04
2020–07

T

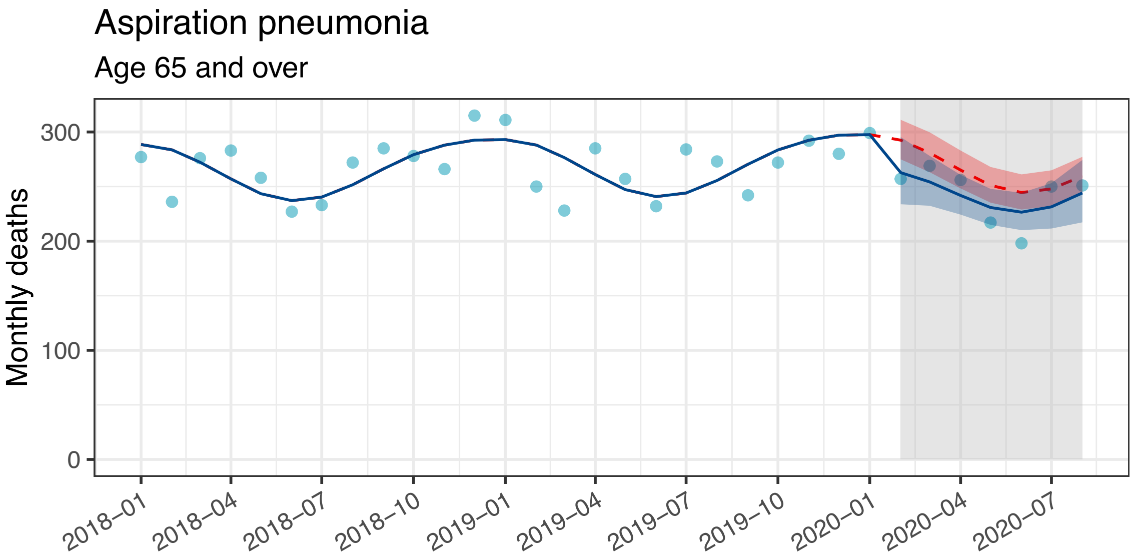

Aspiration pneumonia
Age 65 and over
Monthly deaths
300
200
100
0
2018–01
2018–04
2018–07
2018–10
2019–01
2019–04
2019–07
2019–10
2020–01
2020–04
2020–07

TABLE S2. Estimates and BIC for all models

Outcome: The number of hospitalizations

Disease	Age, years	Pneumonia type	Changepoint	BIC	Δ BIC	Estimated level change rate %	(95% CI)
Pneumonia	≧ 18		Null	-56.51	65.37	-	-
			February	-121.88	0	-24.3	(-32.8 to -14.8)
			March	-110.98	10.9	-26.5	(-35.9 to -15.8)
			April	-105.21	16.67	-32.4	(-41.8 to -21.3)
Pyelonephritis	≧ 18		Null	-171.27	1.2	-	-
			February	-170.77	1.7	-5.4	(-12.8 to 2.6) †
			March	-168.89	3.58	-3.4	(-11.5 to 5.4) †
			April	-172.47	0	-10.1	(-17.8 to -1.6) †
Biliary tract infections	≧ 18		Null	-164.17	0	-	-
			February	-156.36	7.81	-1.1	(-9.7 to 8.3) †
			March	-156.3	7.87	-0.2	(-9.4 to 9.8) †
			April	-156.93	7.24	-4.2	(-13.5 to 6.0) †
Pneumonia	18-64		Null	-29.78	33.85	-	-
			February	-62.34	1.29	-11.6	(-26.7 to 6.7)
			March	-63.32	0.31	-23.2	(-36.9 to -6.6)
			April	-63.63	0	-33.6	(-46.1 to -18.3)
	≧ 65		Null	-55.1	66.76	-	-
			February	-121.86	0	-25.9	(-34.2 to -16.6)
			March	-108.66	13.2	-27.2	(-36.7 to -16.4)
			April	-102.29	19.57	-32.7	(-42.3 to -21.4)
Pneumonia	18-64	Pneumococcal pneumonia	Null	72.48	58.99	-	-
			February	13.49	0	-46.7	(-62.0 to -25.5)
			March	16.84	3.35	-57.1	(-70.2 to -38.2)
			April	14.68	1.19	-70.7	(-79.9 to -57.1)
		Aspiration pneumonia	Null	-59.51	0	-	-
			February	-56.48	3.03	-5.4	(-22.2 to 15.2)
			March	-57.63	1.88	-14.1	(-30.0 to 5.5) †
			April	-59.1	0.41	-21.2	(-36.5 to -2.3) †
		Influenza pneumonia	Null	152.67	2.77	-	-
			February	149.9	0	-77.5	(-91.4 to -41.1)
			March	152.38	2.48	-76.4	(-91.6 to -33.6)
			April	160.07	10.17	-38.8	(-80.9 to 95.6)
	≧ 65	Pneumococcal pneumonia	Null	35.35	69.06	-	-
			February	-33.71	0	-45.2	(-56.6 to -30.8) †
			March	-20.48	13.23	-47.7	(-60.2 to -31.2) †
			April	-13.25	20.46	-54.9	(-66.8 to -38.7) †
		Aspiration pneumonia	Null	-108.22	39.5	-	-
			February	-147.72	0	-14.0	(-21.9 to -5.2)

Disease	Age, years	Pneumonia type	Changepoint	BIC	Δ BIC	Estimated level change rate %	(95% CI)
			March	-140.11	7.61	-12.9	(-21.9 to -2.9) †
			April	-140.15	7.57	-19.3	(-28.1 to -9.4) †
		Influenza pneumonia	Null	187.18	21.73	-	-
			February	165.45	0	-94.2	(-98.0 to -83.0)
			March	173.28	7.83	-93.7	(-98.1 to -78.9)
			April	188.2	22.75	-82.7	(-95.9 to -26.8)

†: A Breusch-Godfrey test for autocorrelation was $P < 0.05$, indicating that the time series have autocorrelation. Confidence interval may be underestimated and thus should be interpreted with caution.

Abbreviation: BIC: Bayesian information criterion; ΔBIC: difference in BIC from the best model; CI, confidence interval

Outcome: The number of 30-day deaths

Disease	Age, years	Pneumonia type	Change point	BIC	ΔBIC	Estimated level change rate %	(95% CI)
Pneumonia	≧ 18		Null	-111.44	10.18	-	-
			February	-121.62	0	-16.1	(-25.5 to -5.5)
			March	-115	6.62	-12.3	(-23.2 to 0.1)
			April	-112.01	9.61	-10.7	(-22.6 to 3.0)
Pyelonephritis	≧ 18		Null	2.49	0	-	-
			February	10.66	8.17	1.1	(-27.2 to 40.5)
			March	10.55	8.06	4.3	(-26.3 to 47.6)
			April	10.44	7.95	5.9	(-26.7 to 52.9)
Biliary tract infections	≧ 18		Null	-30.39	0	-	-
			February	-25.31	5.08	-18.4	(-36.4 to 4.8)
			March	-25.68	4.71	-21.0	(-39.2 to 2.8)
			April	-23.13	7.26	-13.0	(-34.5 to 15.6)
Pneumonia	18-64		Null	44.35	0	-	-
			February	49.71	5.36	42.9	(-8.4 to 122.8)
			March	49.3	4.95	50.4	(-5.8 to 140.1)
			April	48.51	4.16	60.3	(-2.0 to 162.3)
	≧ 65		Null	-109.79	12.71	-	-
			February	-122.5	0	-17.4	(-26.6 to -7.1)
			March	-114.93	7.57	-13.7	(-24.4 to -1.5)
			April	-111.49	11.01	-12.4	(-24.1 to 1.2)
Pneumonia	18-64	Pneumococcal pneumonia	Null	176.44	0	-	-
			February	182.24	5.8	25.6	(-82.9 to 480.7)
			March	182.1	5.66	9.1	(-90.4 to 493.5)
			April	182.11	5.67	-44.5	(-98.9 to 359.9)
		Aspiration pneumonia	Null	316.81	0	-	-
			February	323.65	6.84	27.7	(-32.2 to 129.6)
			March	319.83	3.02	101.9	(9.3 to 258.2)
			April	324.77	7.96	12.5	(-46.9 to 120.3)
	≧ 65	Pneumococcal pneumonia	Null	152.7	11.35	-	-
			February	159.08	0	-40.4	(-59.5 to -12.2)

Disease	Age, years	Pneumonia type	Change point	BIC	ΔBIC	Estimated level change rate %	(95% CI)
			March	158.29	5.9	-31.8	(-55.5 to 4.6)
			April	154.14	7.7	-34.1	(-58.4 to 4.3)
		Aspiration pneumonia	Null	43.03	0	-	-
			February	31.68	3.37	-10.2	(-21.0 to 2.2)
			March	37.58	5.67	-6.9	(-18.9 to 6.9)
			April	39.38	5.07	-10.1	(-22.3 to 4.0)

†: A Breusch-Godfrey test for autocorrelation was $P < 0.05$, indicating that the time series have autocorrelation. Confidence interval may be underestimated and thus should be interpreted with caution.

Abbreviation: BIC: Bayesian information criterion; ΔBIC: difference in BIC from the best model; CI, confidence interval

FIGURE S2. Sensitivity analysis: a saturated seasonality model allowing for fully flexible seasonal trend.

A. Monthly trend in the observed number of pneumonia incidence between September 2015 and August 2020. **B.** Interrupted time-series analysis using a saturated seasonality model. The degree of the Fourier-transformed term was increased from 2 (baseline) to 6 (saturated model). The Fourier-transformed term with a degree 6 has 12 free parameters and captures any recurring pattern as the seasonal trend.

A

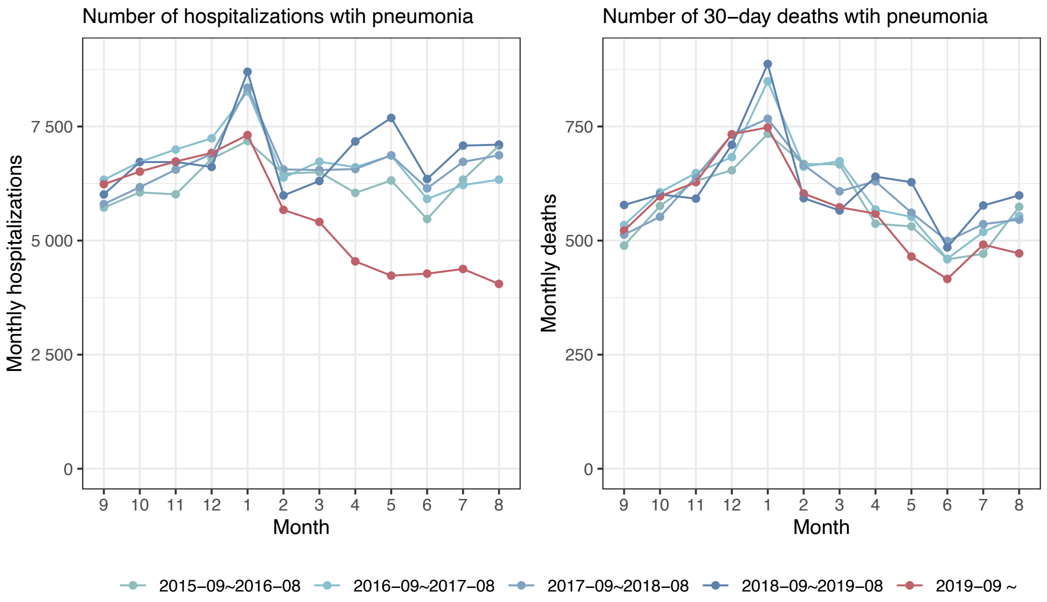

B

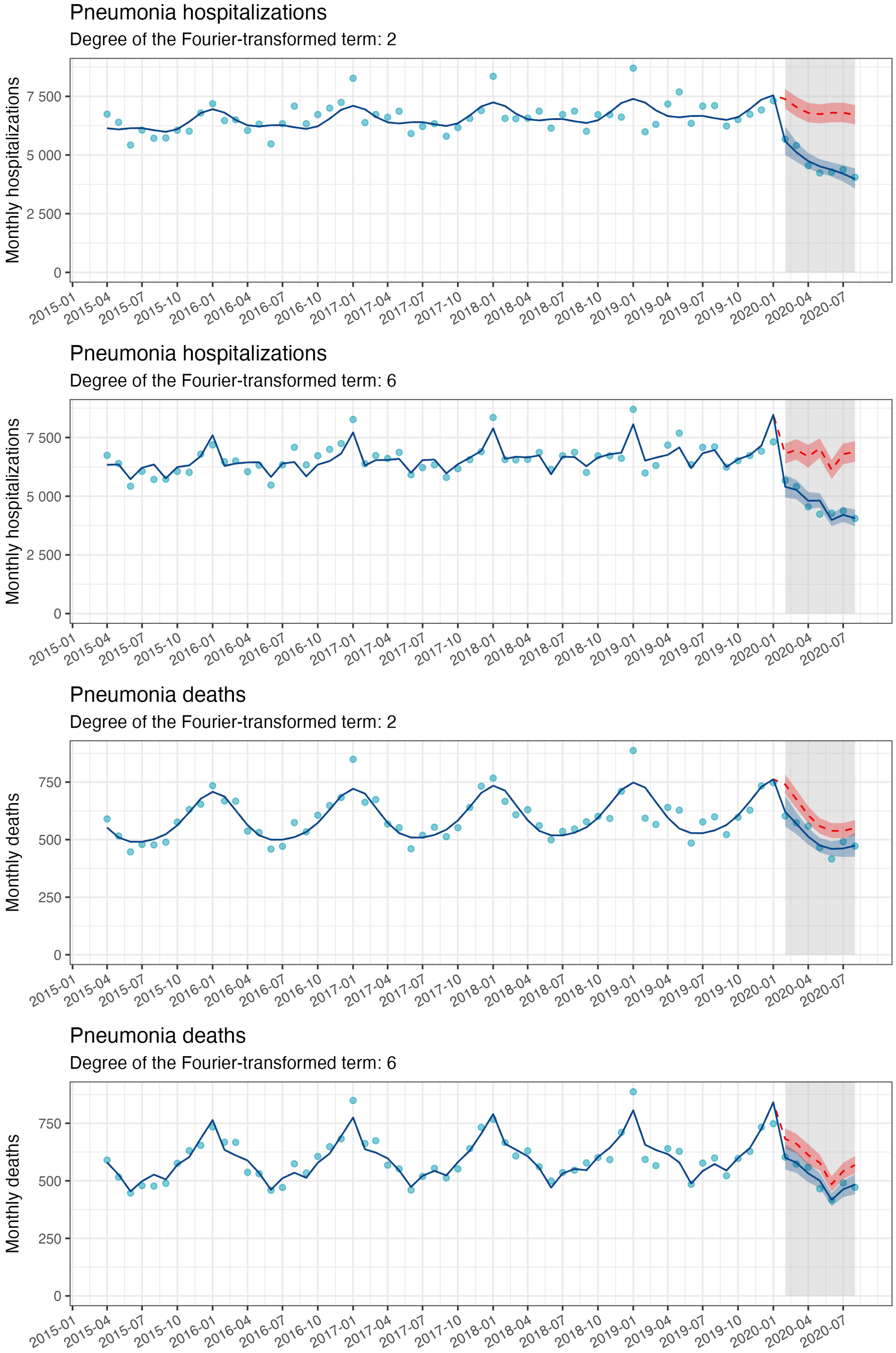
Pneumonia hospitalizations
Degree of the Fourier-transformed term: 2
Monthly hospitalizations
7 500
5 000
2 500
0
Pneumonia hospitalizations
Degree of the Fourier-transformed term: 6
Monthly hospitalizations
Pneumonia deaths
Degree of the Fourier-transformed term: 2
Monthly deaths
750
500
250
0
Pneumonia deaths
Degree of the Fourier-transformed term: 6
Monthly deaths
2015-01
2015-04
2015-07
2015-10
2016-01
2016-04
2016-07
2016-10
2017-01
2017-04
2017-07
2017-10
2018-01
2018-04
2018-07
2018-10
2019-01
2019-04
2019-07
2019-10
2020-01
2020-04
2020-07

TABLE S3. Estimates in the sensitivity analysis with a saturated seasonality model.

	Degree of the Fourier-transformed term	Level change rate in February 2020		
		Estimate %	(95% CI)	P-value
Monthly hospitalizations	2 (baseline)	-24.3	(-32.8 to -14.8)	< 0.001
	6 (saturated model)	-20.7	(-27.7 to -12.9)	< 0.001
Monthly 30-day deaths	2 (baseline)	-16.1	(-25.5 to -5.5)	0.005
	6 (saturated model)	-11.9	(-19.9 to -3.1)	0.010

REFERENCES

1. The Asahi Shimbun Company. Kikuzo II Visual (Japanese). http://database.asahi.com/library2/main/top.php (accessed May 13, 2022).

2. The Yomiuri Shimbun. Yomidas Rekishikan (Japanese). https://database.yomiuri.co.jp/about/rekishikan/ (accessed May13, 2022).

3. World Health Organization. Advice for the public: Coronavirus disease (COVID-19). https://www.who.int/emergencies/diseases/novel-coronavirus-2019/advice-for-public (accessed May 13, 2022).
